# Duplex Sequencing Uncovers Recurrent Low-frequency Cancer-associated Mutations in Infant and Childhood *KMT2A*-rearranged Acute Leukemia

**DOI:** 10.1097/HS9.0000000000000785

**Published:** 2022-09-30

**Authors:** Mattias Pilheden, Louise Ahlgren, Axel Hyrenius-Wittsten, Veronica Gonzalez-Pena, Helena Sturesson, Hanne Vibeke Hansen Marquart, Birgitte Lausen, Anders Castor, Cornelis Jan Pronk, Gisela Barbany, Katja Pokrovskaja Tamm, Linda Fogelstrand, Olli Lohi, Ulrika Norén-Nyström, Johanna Asklin, Yilun Chen, Guangchun Song, Michael Walsh, Jing Ma, Jinghui Zhang, Lao H. Saal, Charles Gawad, Anna K. Hagström-Andersson

**Affiliations:** 1 Division of Clinical Genetics, Department of Laboratory Medicine, Lund University, Lund, Sweden; 2 Division of Pediatric Hematology/Oncology, Stanford University, School of Medicine, Stanford, CA, USA; 3 Department of Clinical Immunology, National University Hospital, Rigshospitalet, Copenhagen, Denmark; 4 Department of Paediatrics and Adolescent Medicine, Rigshospitalet, University of Copenhagen, Denmark; 5 Childhood Cancer Center, Skane University Hospital, Lund, Sweden; 6 Department of Molecular Medicine and Surgery, Karolinska Institutet, Stockholm, Sweden; 7 Department of Oncology and Pathology, Karolinska Institutet, Stockholm, Sweden; 8 Department of Clinical Chemistry, Sahlgrenska University Hospital, Gothenburg, Sweden; 9 Department of Laboratory Medicine, Institute of Biomedicine, University of Gothenburg, Sweden; 10 Tampere Center for Child, Adolescent and Maternal Health Research and Tays Cancer Center, Tampere University and Tampere University Hospital, Tampere, Finland; 11 Department of Clinical Sciences, Pediatrics, Umeå University, Umeå, Sweden; 12 SAGA Diagnostics, Lund, Sweden; 13 Department of Pathology, St. Jude Children’s Research Hospital, Memphis, TN, USA; 14 Department of Computational Biology, St. Jude Children’s Research Hospital, Memphis, TN, USA; 15 Division of Oncology, Department of Clinical Sciences, Lund University, Lund, Sweden; 16 Center for Translational Genomics, Lund University, Lund, Sweden

## Abstract

Infant acute lymphoblastic leukemia (ALL) with *KMT2A*-gene rearrangements (*KMT2A*-r) have few mutations and a poor prognosis. To uncover mutations that are below the detection of standard next-generation sequencing (NGS), a combination of targeted duplex sequencing and NGS was applied on 20 infants and 7 children with *KMT2A*-r ALL, 5 longitudinal and 6 paired relapse samples. Of identified nonsynonymous mutations, 87 had been previously implicated in cancer and targeted genes recurrently altered in *KMT2A*-r leukemia and included mutations in *KRAS*, *NRAS*, *FLT3*, *TP53*, *PIK3CA*, *PAX5*, *PIK3R1*, and *PTPN11*, with infants having fewer such mutations. Of identified cancer-associated mutations, 62% were below the resolution of standard NGS. Only 33 of 87 mutations exceeded 2% of cellular prevalence and most-targeted PI3K/RAS genes (31/33) and typically *KRAS/NRAS*. Five patients only had low-frequency PI3K/RAS mutations without a higher-frequency signaling mutation. Further, drug-resistant clones with *FLT3*^*D835H*^ or *NRAS*^*G13D/G12S*^ mutations that comprised only 0.06% to 0.34% of diagnostic cells, expanded at relapse. Finally, in longitudinal samples, the relapse clone persisted as a minor subclone from diagnosis and through treatment before expanding during the last month of disease. Together, we demonstrate that infant and childhood *KMT2A*-r ALL harbor low-frequency cancer-associated mutations, implying a vast subclonal genetic landscape.

## INTRODUCTION

Acute lymphoblastic leukemia (ALL) in children below 1 year of age, that is, infants, accounts for 2.5% to 5% of pediatric ALL.^1^ Genetic rearrangements of the *KMT2A* gene (previously *MLL*) are present in around 80% of infant ALL and correlate with a poor prognosis.^1,2^ While the overall survival rate of childhood leukemia has improved during recent years, now exceeding 90%,^3,4^ this success has not been translated to *KMT2A*-rearranged (*KMT2A*-r) infant ALL.^1^ Thus, an increased understanding of its pathogenesis combined with novel therapeutic approaches are needed to improve outcome.

We and others have shown that *KMT2A*-r infant ALL has few somatic mutations present in most leukemia cells, with an average of only 1.3 nonsilent mutations.^5,6^ Despite the paucity of major clonal mutations, activating kinase-PI3K/RAS mutations were present in approximately 50%, and may confer a poorer prognosis.^5,7^ About half of the activating mutations identified were subclonal with variant allele frequencies (VAFs) < 0.30 and some patients harbored several activating mutations at varying VAFs, suggesting multiple clones. Further, considering all mutations, infants had a higher fraction of subclonal mutations compared with children above 1 year of age.^5^ This suggests that infant *KMT2A*-r ALL is clonally heterogeneous, which could contribute to its poor prognosis by allowing relapse from a diagnostic subclone.

Conventional next-generation sequencing (NGS) allows detection of mutations down to 0.01 to 0.05 VAF. Identification of lower-frequency variants is not feasible given the error-rate of library preparation and sequencing.^8,9^ Recent developments such as duplex sequencing (DS)^10,11^ that use unique barcodes allowing for error-correction, can depending on the number of cell equivalents and sequencing depth, detect mutations present in 1 × 10^–6^ cells.

Given the high fraction of subclonal mutations in infant ALL, in particular that target the PI3K/RAS-pathway, we here aimed to investigate if low-frequency mutations in those genes are common at diagnosis thereby providing a reservoir of genetically diverse leukemia clones with such mutations. We therefore applied a combination of DS targeting cancer-associated mutations in 28 genes and NGS on diagnostic samples from 20 infant and 7 childhood *KMT2A*-r ALLs, as well as on 5 longitudinal samples during therapy, and 6 paired relapse samples.

## METHODS

### Patients

Diagnostic samples from 20 infant (0–12 months of age) and 7 childhood *KMT2A*-r ALL (1–15 years of age), diagnosed 2007 to 2012, were studied (24 B-precursor, 2 T-cell, and 1 Bilineage leukemia). Samples were assessed for *KMT2A*-rearrangements as part of clinical diagnostics. Six paired diagnostic-relapse samples were analyzed by targeted resequencing. For P28, we also applied DS for personal targets on samples at days 0, 29, 49, 93, 173, and 208. Samples were obtained with informed consent according to the declaration of Helsinki and this study was approved by the local Ethics committee of Lund University, Sweden.

### Panel design

The panel spanned 6328 nucleotides and 71 probes targeting regions in 28 genes that were either recurrently mutated in *KMT2A*-r infant ALL^5^ or were among the 35 most common mutations in “AML,” “ALL,” or “‘B-cell ALL” in the Catalogue of Somatic Variants in Cancer (COSMIC).^12^ The P28 panel contained 55 mutations identified in the diagnostic or relapse sample (day 208) by WGS/WES. Four variants were excluded because the DS VAF did not match the discovery VAF. Oligo probes, barcode-, amplification-, and indexing primers were ordered from IDT (available upon request).

### DNA extraction

DNA was extracted using various standard protocols including Gentra Puregene Blood kit, QIAamp DNA micro-kit, Allprep DNA/RNA/miRNA Universal kit, or Allprep DNA/RNA Mini kit (Qiagen, Hilden, Germany). When extracted from TRIzolReagent (Thermo Fisher Scientific, Waltham, MA), DNA was isolated from the interphase. To the separated interphase, 500 µL extraction buffer (4 M Guanidine thiocyanate, 50 mM Sodium citrate, 1 M Tris) was added, heated for 5 minutes at 55°C, mixed for 10 minutes (inversion board), centrifuged at 12,000 × g for 30 minutes, the water phase was transferred and DNA was precipitated with 100% Isopropanol and washed with 80% ethanol. DNA was further purified by the Phase-lock gel system (QuantaBio, Beverly, MA), using Phenol and Chisam (1/24 Isoamyl alcohol+23/24 Chloroform). The DNA concentration was measured by Qubit (Thermo Fisher).

### Duplex sequencing

DS was performed as described,^11^ with the modifications below. For adapter preparation, primer strand and template strand oligos were ordered annealed as 20 nmol ultramers (IDT, Integrated DNA technologies, Coralville, IA). Two hundred microliter annealed oligos (50 µM) were end-repaired (ER) using 29 units Klenow Fragment (3'→5' exo-), 10 μM dNTP in 1×NEB buffer 2 at 37°C, 1 hour. Adaptors were ethanol-purified and cut as described.^11^

Two hundred fifty nanograms DNA was fragmented, ER and A-tailed (AT) by KAPA HyperPlus (Roche, Basel, Switzerland). To test if reduced input material could be used, P3, P10, and P13 were also run with 100 ng. A dilution of 1:100 was also tried when optimizing family size. With 250 ng of input DNA, the theoretical detection limit is roughly 0.005% (ie, mutation present in 1 of 40,000 cells).

Adaptors were ligated to the DNA by mixing 60 µL ER/AT DNA and 10 µL of annealed adaptors in 10 µL ligation buffer and 10 µL ligase (KAPA HyperPlus), and incubated for 15 minutes at 20°C, transferred to a new tube and cleaned using AMPure XP (Beckman Coulter Inc., Brea, CA) according to the protocol. When dry, cleaned adaptor-library ligation was resuspended in 23 µL EB. A 25-µL reaction PCR (13 cycles, heated lid, 60°C annealing) was run using KAPA HiFi HotStart ReadyMix with 10.5 µL of 1:10 diluted library and 1 µL of each barcode primer (see later). The PCR product was cleaned with AMPure XP and resuspended in 30 µL EB, hybridized to capture oligos and washed using SeqCap EZ Hybridization and Wash Kit (Roche). A second PCR (16 cycles, heated lid, 65°C annealing) was run with 10 µL library and 1.25 µL of each amplification primer (see later). The library was cleaned and capture hybridized as described above. A 100 µL indexing PCR was run with 20 µL bead/capture DNA mix, 50 µL Kapa HiFi HotStart RM, and 5 µL indexing primers. A final AMPure wash was made before sequencing.

### PCR-amplicon resequencing

DS variants with VAF > 0.01 were validated by multiplex-PCR followed by NGS and other captured variants irrespective of VAF were also assessed. Since the resolution of multiplex-PCR is lower than DS, lack of validation for a VAF < 0.01 mutation did not exclude that mutation. Primers were designed using Primer3^13^ and multiplex compatibility by PrimerTK (https://github.com/stjude/PrimerTK) and isPCR (https://github.com/bowhan/kent/tree/master/src/isPcr).

For the paired diagnosis-relapse samples, a multiplex primer-mix were designed for all diagnostic DS variants, followed by resequencing in diagnostic+relapse samples. Primers were purchased from IDT (available upon request). A multiplex-PCR was performed using Qiagen’s Multiplex-PCR kit according to protocol. PCR amplicons were purified using AMPure XP and prepared for sequencing (Nextera XT DNA Sample Preparation and Index Kit, Illumina, San Diego, CA, USA). 2 × 150 bp paired-end sequencing was performed using NextSeq500 or MiSeq (Illumina).

### Bioinformatics analysis

DS Raw reads were transformed to unaligned SAM format using Picard (version: 2.6.0, Broad institute, Cambridge, MA). Reads were collapsed to Single Strand Consensus sequences (SSCS) and Dual Strand Consensus (DCS) using UnifiedConcensusMaker.py with standard parameters (–cutoff 0.7, —minmem 3 —maxmem 200 —rep_filt 9) from the DS software (Version 3.0).^11^ SSCS and DCS reads were aligned using BWA-MEM (version 0.7.15), and Indel realigned (GATK version: 3.6).^14^ Five base pairs from 3' and 5' of each read was trimmed using BamUtils.^15^ Targeted resequencing adapters were trimmed using Trimmomatic (0.32)^16^ and aligned as earlier.

Processed reads were piled up using samtools^17^ mpileup (1.3.1, parameters: -B,-d30000-q55). Quality control revealed that the P28 day 29 sample had low SSCS coverage due to extreme family sizes (largest: 11 564); moreover, most of the reads in 1 region derived to 1 biological fragment. To rescue this sample in the longitudinal analysis, we estimated the VAFs from this timepoint from DCS-BAM files by manual inspection of 8 relapse variants that were also present at diagnosis.

Variant calling was performed by VarScan (2.4.1, DS-parameters: -min-var-freq 0.001, –p-value 0.1, –min-avg-qual 25; P28-parameters: -min-var-freq 0.000001, –min-avg-qual 30; multipex PCR-parameters: –min-var-freq 0.001, –strand-filter 0, –p-value 0.05). To filter alignment artifacts, we demanded that at least 1 mutant read was without soft-clipping and if more than 10 samples had the same mutation, the region was manually assessed. Available WES/WGS data (not shown) was used to add support for DS variants by searching in the BAM-files as described.^18^

Mutations were crosschecked against the Genome Aggregation Database (GnomAD)^19^ to ensure that no variant that failed AC0 or RF in both genome and exome data was present. Variants were also annotated against Exome Aggregation Consortiums (ExAC)^20^ to cross-check for common variants in the population. Mutational patterns^21^ were used to assign COSMIC mutational signatures.^22^

For paired diagnostic-relapse variants, if detected relapse variant was not identified in DCS reads at diagnosis, we manually inspected SSCS reads. This was done for the diagnostic *NRAS*^G13D^ (P3)*, NRAS*^G12S^ (P28), and *FLT3*^D835H^ (P58).

Clonal composition in longitudinal samples (P28) was inferred by assigning mutations to clusters. Mutations clustering together over time were considered to reside in the same clone and at least 2 mutations were required to call a clone.

### Definition of cancer-associated mutations

To define cancer-associated mutations, we required the amino acid change to be present in PeCAN (https://pecan.stjude.cloud/)^23^ or in COSMIC,^15^ classified as “mutation significance tier” 1–3, or that the mutation was reported more than 5 times.

### Digital droplet PCR

Targeted ultradeep mutation detection was performed using SAGAsafe digital PCR (SAGA Diagnostics, Lund, Sweden), as previously described.^24,25^ In brief, SAGAsafe is an enhanced dPCR technology with significantly improved sensitivity and specificity, allowing for quantification of alleles to 0.001% VAF [George et al. manuscript in preparation]. SAGAsafe assays targeting NRAS^Q61H^, NRAS^Q61R^, KRAS^G12C^, and TP53^G245V^ were designed and validated using synthetic positive controls or tumor DNA positive control and at least 360 ng human normal DNA (Promega, Madison, USA). All assays were confirmed to have a lower limit of detection of 0.0044% VAF or better with sufficient input material used. For each mutation assessed, 110 ng of DNA was used.

### Statistical methods

All statistical tests were performed in R (4.0). All 2 group comparisons were performed using the nonparametric Mann-Whitney *U* test.

## RESULTS

### Most low-frequency mutations would go undetected by standard NGS

To gain a more comprehensive insight into the subclonal genetic landscape we performed DS^10,11^ of leukemia-associated mutations in 28 genes on 20 infant (<1 year) and 7 childhood (1 to 15 years) *KMT2A*-r ALL patients, on 5 longitudinal samples during therapy, and 6 paired relapse samples (Suppl. Table S1). The targets included genes in the PI3K/RAS-pathway that are recurrently mutated in infant ALL^5^ and selected genes mutated in acute leukemia (Suppl. Table S2; Methods). The captured regions had a median coverage of 1384-6589X DCS reads (Suppl. Figure S1A–E and Suppl. Tables S2–4).

**Figure 1.**
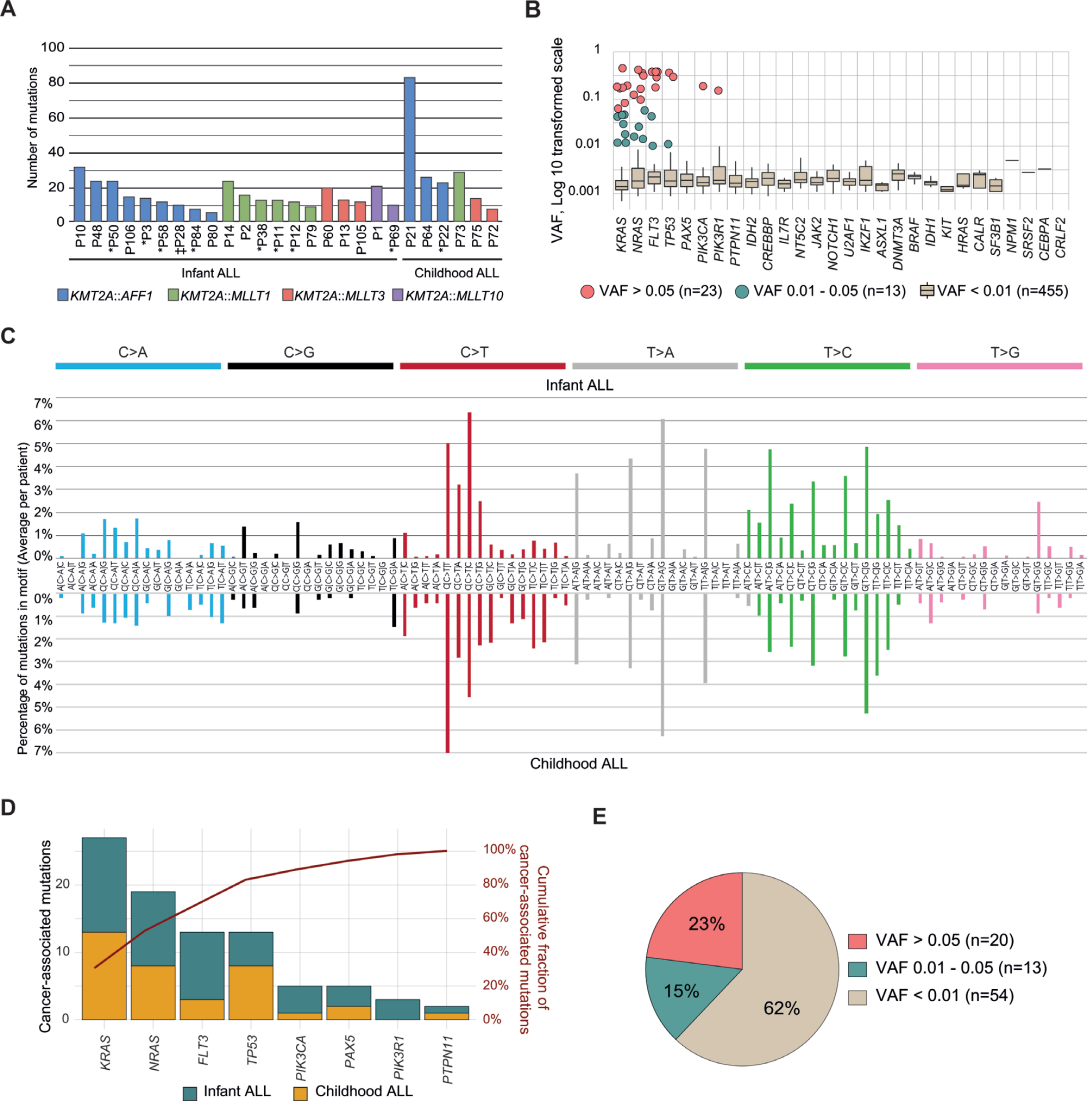
**Overall mutation abundance and genes targeted by cancer-associated mutations.** (A) Number of total mutations in each patient. Bars are colored by the specific *KMT2A*-fusion in the patient. * Relapse; ‡Resistant disease. (B) The VAFs of all silent and nonsilent mutations across the 28 genes, with 94% of mutations having a VAF <0.01 and thus being below the resolution of standard deep sequencing panels. The genes were ordered based on the number of cancer-associated mutations and then the total number of mutations. (C) Average contribution per patient to COSMIC mutational signatures in infant ALL (above) and childhood ALL (below). (D) The number of cancer-associated mutations across the 8 genes affected by such mutations, with all of them targeting genes mutated in *KMT2A*-r leukemia, including *KRAS* (n = 27, in 13 cases), *NRAS* (n = 19, in 11 cases), *FLT3* (n = 13, in 8 cases), *TP53* (n = 13, in 8 cases), *PIK3CA* (n = 5, in 4 cases), *PAX5* (n = 5, in 3 cases), *PIK3R1* (n = 3, in 2 cases) and *PTPN11* (n = 2, 2 cases), The red line shows the cumulative percentage of mutations. (E) Of the 87 cancer-associated mutations, 54 would not have been detected by standard next-generation sequencing (VAF < 0.01), and only 20 would have been identified by whole genome or whole exome sequencing (VAF > 0.05). Another 13 mutations occurred at levels that require panel re-sequencing for identification (VAF 0.01–0.05). VAFs = variant allele frequencies.

Across all bases, 931 mutations were identified and our oldest patient (P87) with T-ALL contributed with half (n = 440) (Suppl. Figure S2A), this patient was removed from further analyses as it would confound downstream statistical associations, giving 491 mutations in 26 samples (nonsynonymous n = 308, synonymous n = 183). A combination of ddPCR, multiplex-PCR followed by NGS and by support from whole exome or whole genome sequencing (WES/WGS) was used to validate 166 mutations (Figure 1A; Suppl. Figure S2B,C; Suppl. Table S5). Infants had a lower number of total mutations than children (average 15 and 31, respectively, *P* = 0.045) (Suppl. Figure S2D; Suppl. Table S5). Only 7.3% of mutations had a VAF > 0.01, thus 93% of mutations comprised less than 2% of cells and were beyond the sensitivity of standard NGS panels (Figure 1B; Suppl. Figure S2E). Apart from 4 genes with 0–1 mutations (*SRSF2*, *CEBPA*, *NPM1*, *CRLF2*), the other genes had 1 mutation every 189–780 bp (Suppl. Figure S2B). The most common base change was C/G→T/A with several mutational processes enriched (Figure 1C; Suppl. Figure S2F).^26^ Combined, most identified mutations were present in such a small fraction of cells that they would go undetected by conventional sequencing strategies.

### All cancer-associated mutations target recurrently mutated genes in *KMT2A*-r ALL

Of identified mutations, 87 occurred at sites that had been previously implicated in leukemia or cancer as determined by the mutation being present in PeCan^23^ or classified as tier 1–3 in COSMIC,^12^ and affected 8 of the 28 investigated genes (Figure 1D; Suppl. Tables S5 and S6, see Methods for a definition of cancer-associated mutations). Importantly, the 20 genes lacking cancer-associated mutations included genes that are rarely or never mutated in *KMT2A*-r ALL (Suppl. Table S2).

Focusing on the 87 cancer-associated mutations which affected 21/26 cases, all targeted genes previously found mutated in *KMT2A*-r leukemia, and typically PI3K/RAS-pathway genes and included *KRAS* (n = 27, 13 cases), *NRAS* (n = 19, 11 cases), *FLT3* (n = 13, 8 cases), *TP53* (n = 13, 8 cases), *PIK3CA* (n = 5, 4 cases), *PAX5* (n = 5, 3 cases), *PIK3R1* (n = 3, 2 cases), and *PTPN11* (n = 2, 2 cases) (Figure 1D; Suppl. Figure S3A,B and Suppl. Table S6).^5,27^ Infants had fewer mutations than children (average 2.6 and 6.0, respectively, *P* = 0.042), with 5 infants lacking cancer-associated mutations (Suppl. Figure S3C,D and Suppl. Tables S5 and S6). When comparing infants that remained in remission to those who relapsed, no correlation to the number of cancer-associated mutations could be seen (*P* = 0.164).

**Figure 2.**
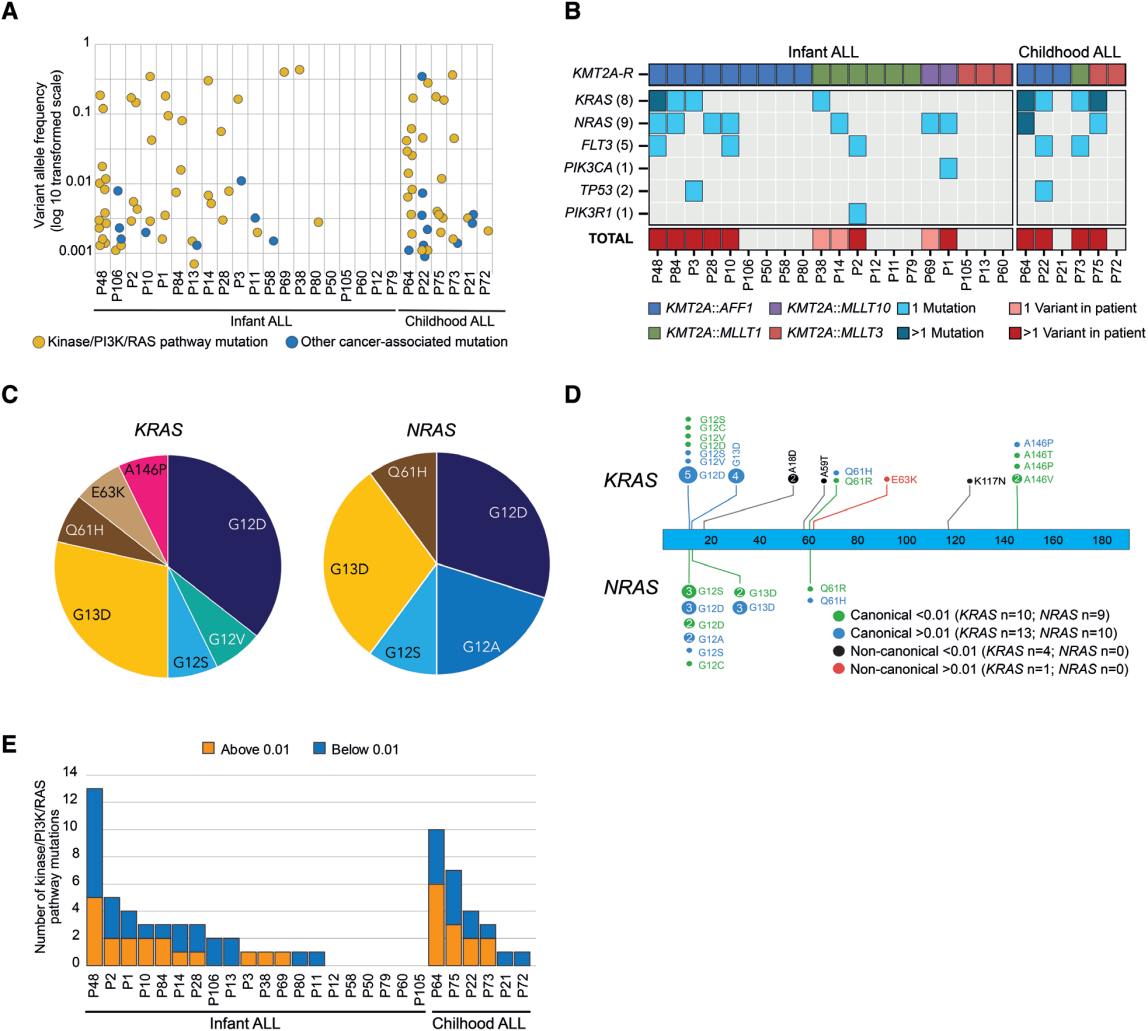
**Kinase-PI3K/RAS mutations were present in a higher fraction of cells.** (A) Distribution of allele frequencies in the 87 cancer-associated mutations in the patients. (B) Heat-map of the 33 mutations with VAF > 0.01 with light blue denoting 1 mutation and dark blue more than 1 mutation in the gene. As many as 24 of the 33 mutations (73%) affected *KRAS* or *NRAS.* The first block shows infants and the second block children. Patients are first sorted on the *KMT2A*-r (top panel) and secondly on if they have a VAF > 0.01 mutation. One mutation is denoted in bottom panel with pink, and 2 or more mutations in red. (C) Of the 24 *KRAS/NRAS* mutations with VAF > 0.01, 23 affected canonical amino acids in *KRAS*/*NRAS* with 83% targeting G12 or G13 and resulting in G12D/G13D. (D) Protein paint showing all identified mutations in *KRAS* and *NRAS* and their respective VAFs with green denoting canonical mutations below a VAF of 0.01 and blue above a VAF of 0.01. Noncanonical mutations below a VAF of 0.01 is denoted in black and in red if above a VAF of 0.01. (E) Number of mutations in kinase-PI3K/RAS-pathway genes in each patient. Blue denotes number of mutations below a VAF of 0.01 and orange denotes mutations above a VAF of 0.01.

Of the 87 mutations, 33 were estimated to be present in clones that exceeded 2% of the cells in each sample (VAFs > 0.01) (Figure 1E and 2A; Suppl. Tables S5 and S6). Six of these 33 higher-frequency mutations resided in the major leukemia clone (VAF > 0.3), 14 mutations were subclonal but in the range of detection with WGS/WES (VAF 0.05–0.3), and the remaining 13 mutations would only have been reliably identified by deep sequencing panels (VAF 0.01–0.05) (Suppl. Figure S3E and Suppl. Table S6). Thus, 62% of the cancer-associated mutations were below detection of standard NGS and only 7% were present in the major leukemic clone. Combined, DS uncovered low-frequency cancer-associated mutations that targeted genes recurrently mutated in *KMT2A*-r leukemia.

### PI3K/RAS mutations were present in larger fractions of cells

Of the 33 cancer-associated mutations that were present in more than 2% of cells, 31 targeted PI3K/RAS genes, a category of genes that is often mutated in *KMT2A*-r infant ALL.^5,27^ The mutations included *KRAS* (n = 14, 8 cases), *NRAS* (n = 10, 9 cases), *FLT3* (n = 5, 5 cases), *PIK3CA* (n = 1) and *PIK3R1* (n = 1) (Figure 2B; Suppl. Table S6). Importantly, 39% of these mutations would not be detected by WGS/WES. The frequency of PI3K/RAS-mutations was similar among infants and children (50% versus 67%) and 74% affected canonical amino acids in *KRAS* (n = 13) and *NRAS* (n = 10) with 87% targeting G12/G13 (Figure 2C,D; Suppl. Figure S4A and Suppl. Tables S6 and S7). *KRAS/NRAS* mutations were found in 45% of infant and 67% of childhood cases with 10% and 33%, respectively, having both genes mutated (Suppl. Figure S4B and Suppl. Table S7).

Including all cancer-associated PI3K/RAS-mutations regardless of VAF (n = 69), 70% of the infants and all children carried at least 1 mutation (average infant: 2.2, range 0–13 and children: 5.1, range 1–10) and when several mutations were present, the VAFs were variable, consistent with the presence of multiple subclones (Figure 2A,E; Suppl. Tables S6 and S7). There was no difference in average VAF between infants and children (*P* = 0.87) (Suppl. Figure S4C). Given the high number of activating mutations in *KMT2A*-r leukemia overall, it is noteworthy that they do not necessarily cause clonal expansion, as 55% had an allele frequency <0.01 (Figure 2A; Suppl. Table S7). Further, 5 patients (19%) only had low-frequency mutations without a concurrent higher-frequency signaling mutation by DS or WGS/WES. Thus, most mutations in clones that comprised >2% of cells targeted *FLT3* or PI3K/RAS-genes, consistent with them being cooperating lesions to the *KMT2A*-r and having a selective advantage.^28^ However, these mutations did not always cause clonal expansion as they often were found in only a small fraction of cells.

### Low-frequency drug-resistant clones expand at relapse

**Figure 3.**
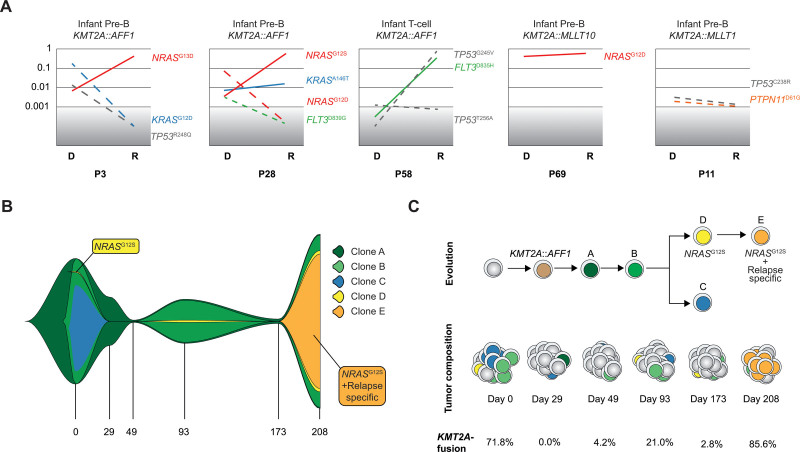
**Clonal evolution from diagnosis to relapse and across therapy.** (A) Variant allele frequency change in cancer-associated mutations from diagnose (DS-date) to relapse (targeted sequencing data) in 5 of the 6 patients with a relapse sample. Patient 12 had no cancer-associated mutations and is not shown. Red: *NRAS* mutation; Blue: *KRAS* mutation; Green: *FLT3* mutation; Gray: *TP53* mutation; Orange: *PTPN11* mutation. Dashed line: mutation is not detected in one of the paired samples. Gray area denotes detection limit of the targeted sequencing. Scale is log 10 transformed (B) FISH-plot showing the clonal evolution in patient 28 with the founding clone “A” in dark green, the major diagnostic clone “B” in dark green, clone “C” in blue, and finally clone “D” in yellow containing *NRAS*^G12S^ which took over in a selective sweep at relapse and had at that time acquired additional mutations, defining clone “E” (orange). (C) Schematic view of clonal evolution and tumor composition in patient 28. The *KMT2A*-r clone in light brown denotes the first event and clone A and B, the first 2 clones before branching. Clone C was lost along treatment, and clone D was seen as a small subclone at diagnosis and through treatment and the last month before relapse, it evolved and accumulated additional mutations that were only detected at relapse (clone E).

As low-frequency mutations may represent drug-resistant clones, we performed multiplex-PCR followed by NGS based on our DS results on 6 paired diagnose-relapse samples. We studied all diagnostic cancer-associated variants for these 6 patients in all 12 samples (n = 20, 0–7 variants/patient, Suppl. Table S6). In 3 patients, minor diagnostic clones with *FLT3*^*D835H*^ (P58) or *NRAS*^*G13D/G12S*^ (P3, P28) mutations at VAF 0.0003–0.0017, expanded at relapse (Figure 3A, Suppl. Table S6). Two of these patients also had higher-frequency diagnostic *KRAS/NRAS* mutations that were lost at relapse (P3, P28). Further, P28 had a *KRAS*^A146T^, which never surpassed 4% of cells and P3 had a diagnostic *TP53*^R248Q^ in 2% of cells which was lost at relapse. The relapse from P58 had a *TP53*^G245V^ at VAF 0.75, indicating allelic imbalance, in addition to the *FLT3*^D835H^ (VAF 0.25). In contrast to the *FLT3*-mutation, both DS and ddPCR failed to detect the *TP53*^G245V^ at diagnosis (ddPCR detection limit VAF 0.00009), suggesting that it might be gained during treatment. P69 had a major diagnostic *NRAS*^G12D^ that was maintained, P11 had low-frequency *PTPN11*^D61G^ and *TP53*^C238R^ which were lost at relapse, and in P12, no cancer-associated mutations were detected (Figure 3A; Suppl. Table S6).^29^ The paired relapse of P11/P12 has not been subjected to WGS/WES but in line with our DS data, the diagnostic samples lacked higher-frequency PI3K/RAS-mutations. Combined, in 3 out of 6 patients, diagnostic low-frequency subclones with *FLT3*^*D835H*^ or *NRAS*^*G13D/G12S*^ expanded at relapse, in line with them being selected for during chemotherapy.

### Clonal evolution in serial samples reveals clone-specific response kinetics

To determine the rise and fall of leukemia clones across therapy, we designed a patient-specific panel from prior WGS/WES at diagnosis and at day 208, and performed DS at diagnosis, on samples from days 29, 49, 93, 173, and 208 (Suppl. Table S8). This patient had a *KMT2A*::*AFF1* and resistant disease, with 0% to 86% *KMT2A*-r cells by fluorescence in situ hybridization (FISH) across the time points.

Longitudinal DS revealed at least 4 leukemia clones at diagnosis; the founding clone (clone A) and 3 subclones (clones B, C, D) (Figure 3B,C; Suppl. Figure S5A–G. The major diagnostic clone (B) was lost at day 29 when no *KMT2A*-R cells were identified by FISH; however, at day 49, it started to expand and, at day 93, it was the major clone again. After 208 days, a diagnostic subclone with an *NRAS*^G12S^ (D) that constituted 0.22% of the major diagnostic clone, took over in a selective sweep, and during expansion, it accumulated mutations that were detected at relapse only (E) (Figure 3B,C; Suppl. Figure S5E and Suppl. Tables S8 and S9). Thus, the relapse clone was found in up to 3% of the major clone across treatment and did not expand until the last month of disease. The second subclone (C) was detected in 56% of the major diagnostic clone and during treatment it never surpassed 0.02% and was lost after day 93, thus the leukemia displayed a pattern of branching evolution (Figure 3B,C; Suppl. Figure S5C and Suppl. Tables S8 and S9). Combined, DS gave insight into the relationship between clones and how they evolved and showed that the relapse clone was present in a very small fraction of the cells through the disease.

## DISCUSSION

We currently lack an accurate estimation of the genetic heterogeneity in *KMT2A*-rearranged leukemias, as well as how that genetic diversity impacts an individual patient’s response to therapy, and ultimately, to the risk of relapse and long-term outcome. Herein, we investigated the presence of cancer-associated mutations, and our results demonstrate that most mutations reside in such a small fraction of cells that they would not be detected by conventional NGS. One model to explain this finding is a large number of leukemic clones with cancer-associated mutations early in leukemogenesis, and that during clonal evolution before diagnosis, a small subset of those clones gets selected for. Alternatively, some of these mutations may not occur in the right mutational, cellular, or environmental contexts for clonal expansion, including the possibility that they reside in normal cells.

Most identified nonsilent mutations had not been associated with leukemia or cancer before and are likely not contributing to leukemogenesis, although we cannot exclude rare variants with a functional impact. The cancer-associated mutations targeted genes that are recurrently mutated in *KMT2A*-r ALL, including PI3K/RAS-genes, *TP53* and *PAX5*, in line with identified mutations likely being biologically relevant.^5,27^ By contrast, the 20 genes that lacked cancer-associated mutations are typically not mutated in *KMT2A*-r leukemia. Further, infants had fewer cancer-associated mutations than children analogous to them having fewer somatic mutations overall.^5^

NGS studies have shown that multiple RAS-pathway mutations with different VAFs within the same patient are common at diagnosis, indicating clonal heterogeneity.^5,30,31^ Herein, consistent with RAS-mutations being cooperating lesions in *KMT2A*-r leukemogenesis,^28^ most cancer-associated mutations targeted such genes. However, more than half of those mutations were found in clones so small that they would not be detected by standard deep sequencing. This suggests an array of cells with individual RAS-pathway mutations and that *KMT2A*-r leukemia is more genetically heterogeneous than previously known. This also raises a question as to why these clones are not expanding as 19% of patients only had low-frequency RAS-mutations without a concurrent higher-frequency mutation. PI3K/RAS-mutations are associated with an average younger age at diagnosis in patients with *KMT2A::AFF1* thereby likely affecting disease latency.^5^ In agreement, *KMT2A*-fusions cause leukemia in mouse models, with cooperating RAS-mutations shortening time to leukemia onset.^28,32–36^ Given that our patients carried small populations with such mutations, either they happened recently in time, or occurred in the wrong context for clonal expansion, including the possibility that they reside in nonmalignant cells. It is also possible that these clones contribute to the cancer microenvironment in a more complex mechanism that is not dependent on clonal dominance.

Minor diagnostic clones with *FLT3*^*D835H*^ or *NRAS*^*G13D/G12S*^ that comprised 0.06% to 0.34% of cells expanded at relapse in 3 patients, in line with them being selected for during treatment.^7,37–40^ Thus, if a subclonal RAS-pathway mutation is present and occur in the right context, it might survive treatment and contribute to relapse. The relapse from P58 had both a *FLT3*^D835H^ and a *TP53*^G245V^ but in contrast to the *FLT3*^D835H^ we were unable to detect *TP53*^G245V^ at diagnosis, suggesting that it was either gained during treatment, or if present at diagnosis, below the level of detection of both DS and ddPCR or due to limited input genome copies. We did detect low-frequency *TP53* mutations at diagnosis, including the *TP53*^R248Q^ (n = 2) which has been shown to be enriched at relapse in ALL.^41^ The 2 patients with diagnostic *TP53*^R248Q^ relapsed, P22 had a testis relapse from which we had no material, but in the other patient (P3) the *TP53*^R248Q^ was lost at relapse and instead a clone with *NRAS*^*G13D*^ expanded (Figure 3A). Our ability to detect mutations is increasing with sensitive technologies, and although they could represent drug-resistant subclones as demonstrated above, they do not always cause relapse since low-frequency driver mutations were seen also in patients that remained in remission. Thus, it is difficult to predict the risk of relapse and long-term outcome based solely on this information, however, as more studies dissecting drug-resistance are emerging, new ways to translate this information to draw clinical insights could emerge.

Our longitudinal analysis provided insight into clonal dynamics during treatment, by demonstrating when in time clones were gained or lost, and identified the leukemia cells across treatment. At day 29 and 49, the fraction of leukemic cells was low, limiting the accuracy of the estimated VAFs at these time points. The same also applies for small subclones, and more genetic material and deeper sequencing would be needed to increase resolution further. Notably, the diagnostic *NRAS*^*G12S*^-subclone that took over in P28 was dormant for 200 days, comprising at the most 3.1% of the leukemia cells during treatment and it expanded over 35 days in a selective sweep.^37^ The rapid expansion of that particular subclone may be connected to a treatment change, to genetic, epigenetic, or microenvironmental changes, and highlight how quickly a subclone can expand to clonal dominance.

Taken together, our data provide new insights into the biology of *KMT2A*-r infant and childhood ALL by showing that the subclonal genetic landscape is more diverse than previously anticipated. The presence of known cancer-associated mutations that do not become dominant at diagnosis or after treatment suggest other factors, such as the cell state,^42^ the microenvironment, or that the combination of mutations is important for clonal selection. Further, predicting risk of relapse based on diagnostic low-frequency mutations may be difficult but instead, the detection of driver mutations along treatment may add more clinically useful information. However, given the high number of low-frequency cancer-associated mutations in each patient, residual treatment-resistant clones with these mutations may cause relapse. Thus, higher-resolution studies such as single-cell RNA and DNA and epigenomic sequencing are likely needed to shed light on the cellular state and how co-occurring mutations cooperate to create phenotypes that result in clonal selection and treatment resistance, and ultimately, patient outcomes.

## ACKNOWLEDGMENTS

We thank the SciLifeLab nodes Center for Translational Genomics, Lund University and Clinical Genomics Lund, and SNP&SEQ Technology Platform, Uppsala, for providing sequencing service.

## AUTHOR CONTRIBUTIONS

MP and AKHA designed experiments; MP, LA, and HS performed experiments; AH-W prepared patient samples; MP performed computational data analyses and statistical analyses; MP, MPW, JM, GS, and AKHA analyzed sequencing data; HVHM, BL, AC, CJP, GB, KPT, LF, OL, and UN-N provided annotated patient samples; JA, YC, and LHS performed ddPCR; JZ developed WGS/WES pipelines and led the analyses; CG and VG optimized the DS protocol; All authors performed critical reading of the manuscript; MP and AKHA wrote the article.

## DISCLOSURES

The authors declare no conflicts of interest.

## SOURCES OF FUNDING

This work was supported by The Swedish Childhood Cancer Fund, The Swedish Cancer Society, The Swedish Research Council, The Knut and Alice Wallenberg Foundation, The Crafoord Foundation, The Per-Eric and Ulla Schyberg Foundation, The Nilsson-Ehle Donations, Governmental Funding of Clinical Research within the National Health Service.

## Supplementary Material


