# Supplementary material for: Duplex Sequencing Uncovers Recurrent Low-frequency Cancer-associated Mutations in Infant and Childhood KMT2A-rearranged Acute Leukemia
Source: Hemasphere. 2022 Sep 30;6(10):e785. doi: 10.1097/HS9.0000000000000785 (PMC9529062; doi:10.1097/HS9.0000000000000785)
Supplement: Supplementary file 2 [file hs9-6-e785-s002.docx]

**
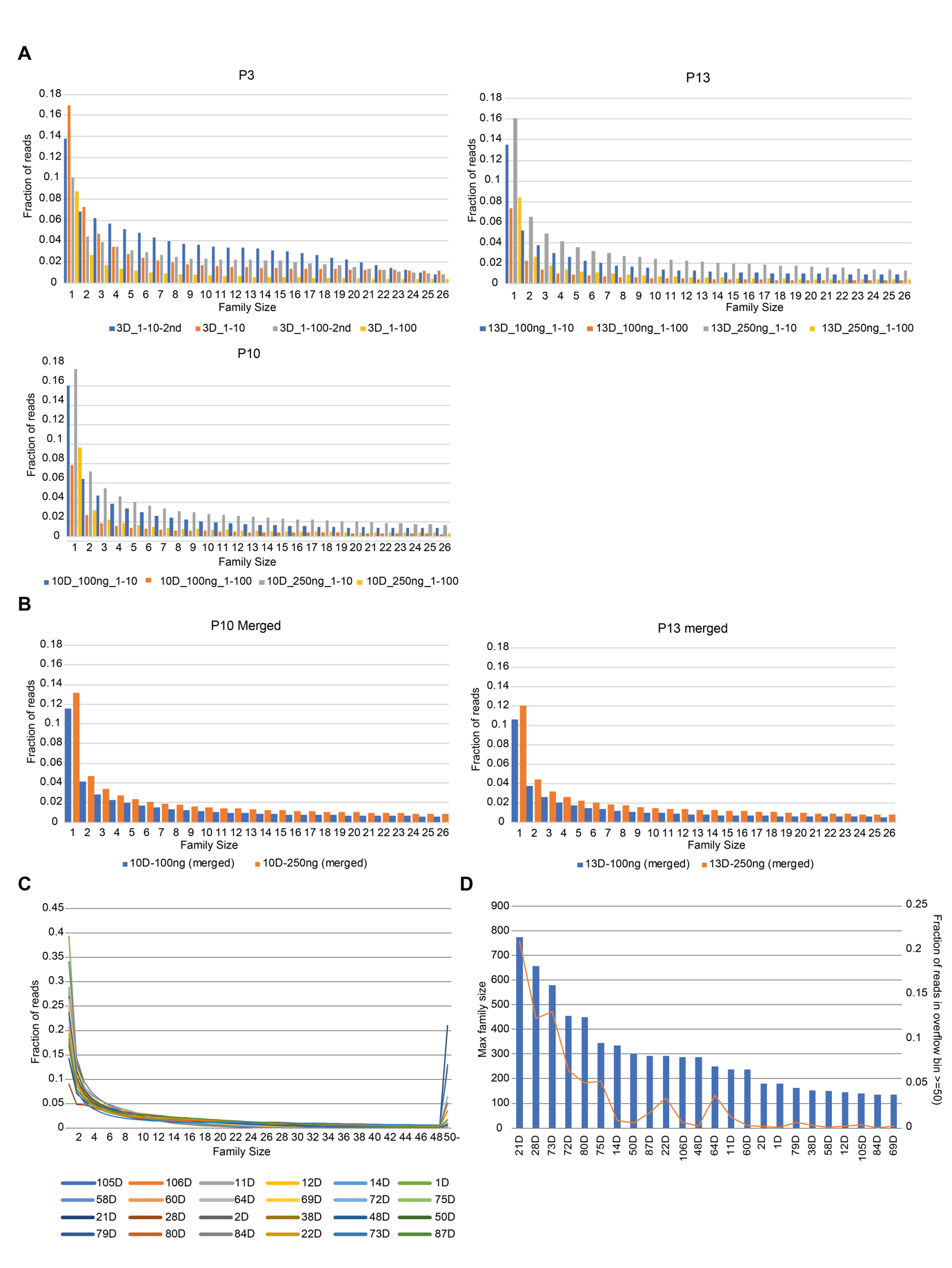
Supplemental Figure 1. Optimization and quality control. (A)** Five sample from three patients were tagmented. To ensure a good representation of biological templates, 250 ng of genomic DNA was used as input in the DS, roughly correlating to 80 000 templates (i.e. chromosomes) from each sample. To test the feasibility to use less material, two libraries with 100 ng (30 000 templates) were also made. After the first PCR, two 250 ng samples and both 100 ng samples were diluted 1:10 and 1:100 to reduce the representation of amplified templates, while one 250 ng sample was kept undiluted. In the undiluted samples, a majority (86%) of the reads makes up a family of size of 2 or less (orphans reads), leaving no room for error correction. Diluting the samples 1:100 reduces orphan reads (19%) but at the same time increasing the median family size, while a dilution factor of 10 keeps the orphans low (37%) while maintaining a low median family size. Higher amounts of input material also increased median family size. (**B**) Sample from patient 10 after merging data from diluted samples. (**C**) Sample from patient 13 after merging data. (**D**) Family size distribution of all other diagnostic samples. Peak family size for all samples is one, indicative of additional sequencing can be made to increase depth. Samples from patient 21, 73 and 28 had more than 10% of reads in big families. (**E**) Bar chart showing the largest family in the rest of the diagnostic samples. Overlayed line graph shows the fraction of reads with family size > 50. Large fraction of reads in big families indicate more sequencing will make families larger instead of increasing depth.

**
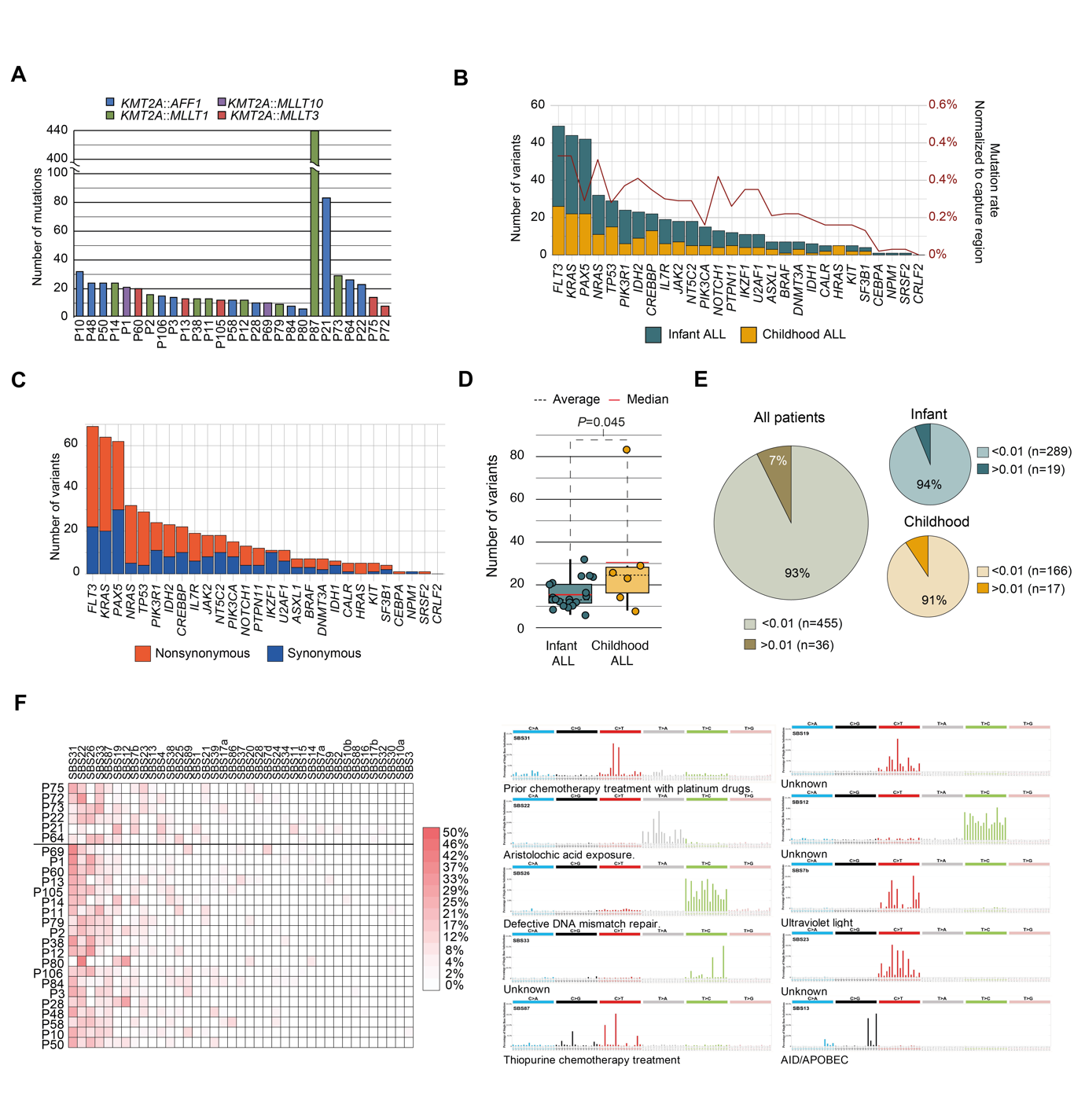
Supplemental Figure 2. The characteristics of mutations in the target region.** (**A**) Number of mutations in each patient. Bars are colored by the specific *KMT2A* fusion in the patient. (**B**) Number of mutations per gene with infant ALL in green and childhood ALL in yellow. The red line shows the capture region-sized normalized value, expressed as the percentage of mutations per base in the captured region in all patients. Most genes (24/28) had one mutation every 189-780 base pairs. The four least mutated genes (*NPM1*, *SRSF2*, *CEBPA* and *CRLF2)* had less than one mutation every 1000 base pairs. (**C**) Number of silent and non-silent mutations in the targeted genes and regions. (**D**) Number of mutations in infants and children. (**E**) Fraction of mutations at > VAF 0.01 and < VAF 0.01, showing that most mutations (96.2%) were below the detection limit of conventional deep sequencing. (**F**) Left: Contribution of each COSMIC mutational signatures in all samples. Right: Top ten mutational signatures.

**
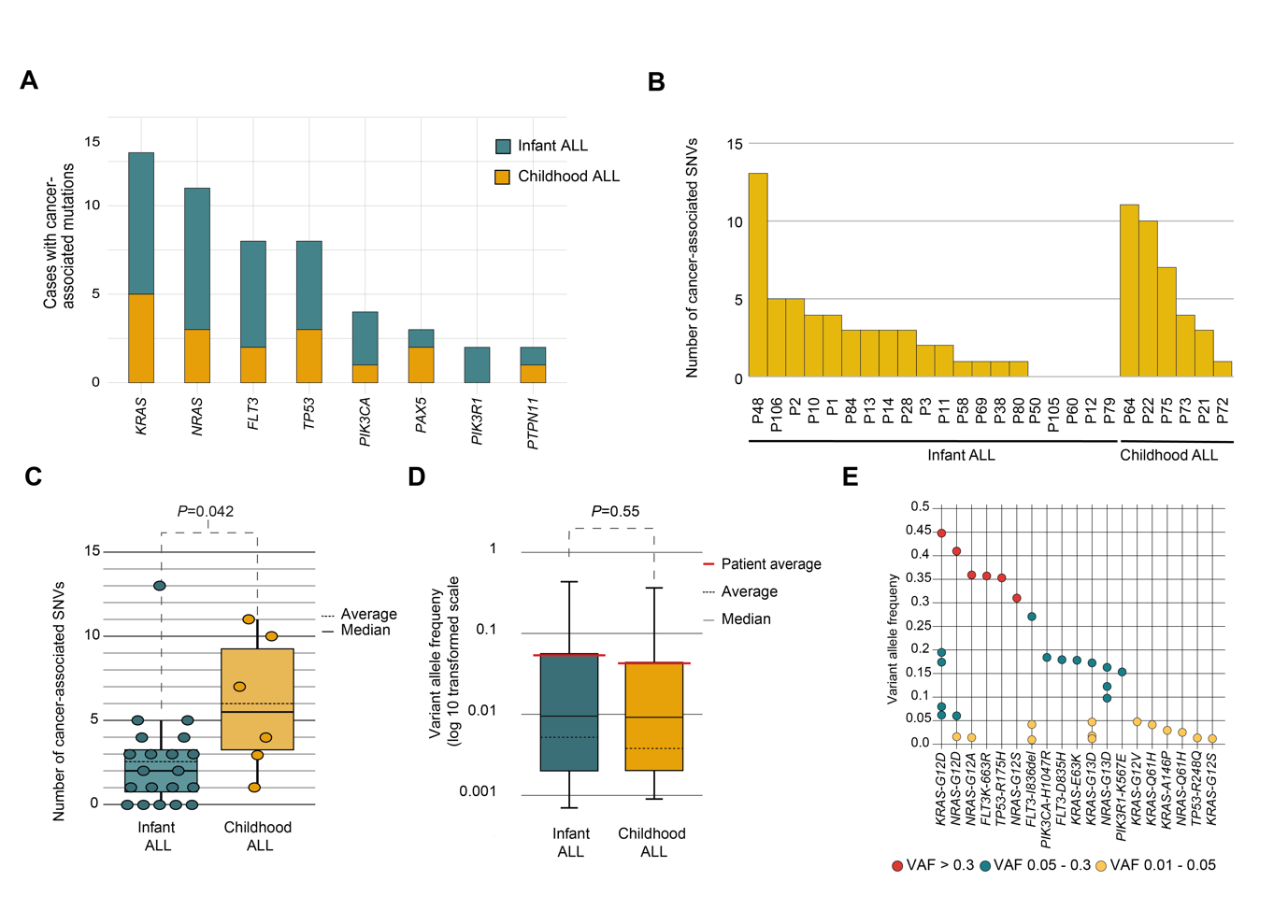
Supplemental Figure 3. Distribution of VAFs and number of cancer-associated mutations.** (**A**) The number of cases with cancer-associated mutation across the 8 genes with such mutations, *KRAS* (13 cases), *NRAS* (11 cases), *FLT3* (8 cases), *TP53* (8 cases), *PIK3CA* (4 cases), *PAX5* (3 cases), *PIK3R1* (2 cases) and *PTPN11* (2 cases) (**B**) Number of cancer-associated mutations in the infant-ALL and childhood ALL cohorts regardless of VAF. (**C**) Boxplot with number of cancer-associated mutations in the infant and childhood ALL cases regardless of VAF. Mann-Whitney U test show significant difference between groups *P*=0.042 **(D)** Boxplot of the distribution of allele frequencies of the 87 cancer-associated mutations the infant-ALL and childhood ALL groups. Mann-Whitney U test show non-significant difference between groups (*P*=0.55). (**D**) The VAFs of the 33 mutations present in at least 2% of the cells.

**
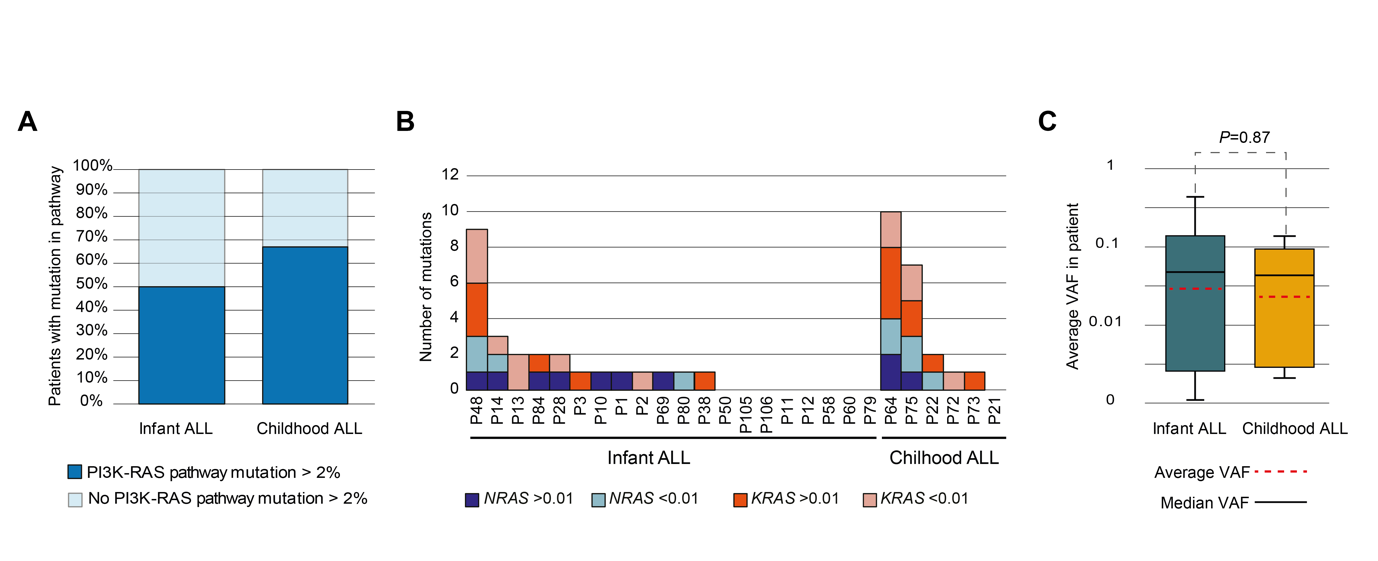
Supplemental Figure 4. Distribution of VAFs and number of mutations in kinase-PI3K/RAS pathway genes.** (**A**) The fraction of infants and children with a VAF>0.01 mutation in the PI3K-RAS pathway with 50% (10/20) and 67% (4/6) of the infants and children, respectively, carrying such a mutation (**B**) Number of canonical mutations in *NRAS* and *KRAS* with VAF above and below 0.01 in all patients. (**C**) Average VAF of kinase-PI3K-RAS pathway mutations in all patients. Average VAF in infants: 0.105 and children: 0.052

**
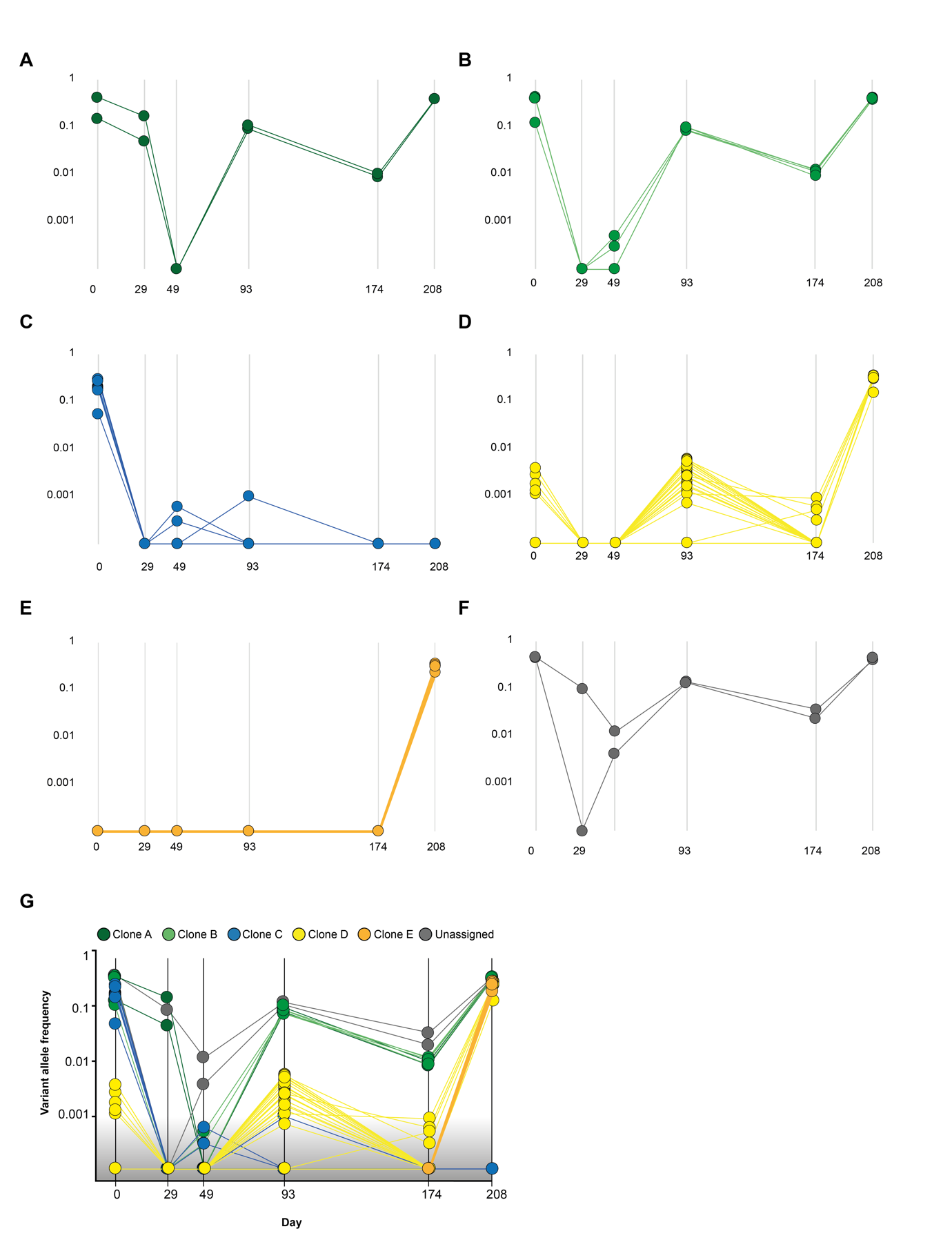
Supplemental Figure 5. Leukemia size and clonal composition over time in patient 28.** (**A**) Change in allele frequencies over treatment in the 2 mutations defining founding clone ‘A’. (**B**) Change in allele frequencies over treatment in the 4 mutations defining founding clone ‘B’. (**C**) Change in allele frequencies over treatment in the 11 mutations defining falling clone ‘C’. (**D**) Change in allele frequencies over treatment in the 31 mutations defining relapsing clone ‘D’. (**E**) Change in allele frequencies over treatment in the 10 mutations defining relapse only clone ‘E’. (**F**) Change in allele frequency over treatment in the 2 mutations that could not be assigned to a clone. (**G**) Line-graph showing the clonal composition along treatment in patient 28 with the founding clone ‘A’ in dark green, the major diagnostic clone ‘B’ in dark green, clone ‘C’ in blue, and finally clone ‘D’ in yellow containing *NRAS*^G12S^ which took over in a selective sweep at relapse and had at that time acquired additional mutations, defining clone ‘E’ (orange). Gray area denotes the diminishing accuracy of the assay, with lower VAF. ***Note****:* At least two mutations were required to infer a clone. VAFs below 0.001 were deemed to be close to the detection limit so outlier mutations showing a different VAF as compared to other mutations in that clone was not reacted upon.
